# AC Electrokinetics of Salt-Free Multilayered Polymer-Grafted Particles

**DOI:** 10.3390/polym12092097

**Published:** 2020-09-15

**Authors:** Silvia Ahualli, Sara Bermúdez, Félix Carrique, María L. Jiménez, Ángel V. Delgado

**Affiliations:** 1 Department of Applied Physics, School of Sciences, University of Granada, 18071 Granada, Spain; sarabermudez@correo.ugr.es (S.B.); jimenezo@ugr.es (M.L.J.); 2 Department of Applied Physics I, School of Sciences, University of Málaga, 23071 Málaga, Spain; carrique@uma.es

**Keywords:** dielectric dispersion, dynamic mobility, layer-by-layer, multi-layer coating, polymer grafting, salt-free, soft particles

## Abstract

Interest in the electrical properties of the interface between soft (or polymer-grafted) nanoparticles and solutions is considerable. Of particular significance is the case of polyelectrolyte-coated particles, mainly taking into account that the layer-by-layer procedure allows the control of the thickness and permeability of the layer, and the overall charge of the coated particle. Like in simpler systems, electrokinetic determinations in AC fields (including dielectric dispersion in the 1 kHz–1 MHz frequency range and dynamic electrophoresis by electroacoustic methods in the 1–18 MHz range) provide a large amount of information about the physics of the interface. Different models have dealt with the electrokinetics of particles coated by a single polymer layer, but studies regarding multi-layered particles are far scarcer. This is even more significant in the case of so-called salt-free systems; ideally, the only charges existing in this case consist of the charge in the layer(s) and the core particle itself, and their corresponding countercharges, with no other ions added. The aims of this paper are as follows: (i) the elaboration of a model for the evaluation of the electrokinetics of multi-grafted polymer particles in the presence of alternating electric fields, in dispersion media where no salts are added; (ii) to carry out an experimental evaluation of the frequency dependence of the dynamic (or AC) electrophoretic mobility and the dielectric permittivity of suspensions of polystyrene latex spherical particles coated with successive layers of cationic, anionic, and neutral polymers; and (iii) finally, to perform a comparison between predictions and experimental results, so that it can be demonstrated that the electrokinetic analysis is a useful tool for the in situ characterization of multilayered particles.

## 1. Introduction

Although the investigation of the properties of nanoparticles is nowadays loaded with interest from the academic point of view (they can be considered in the crossing of fields of chemistry (their synthesis), materials science(their engineering and use as building blocks) or physics (the explanation of their behaviour)), interest in them has grown exponentially when more fields of application have been disclosed [1]. The generation of such applications has taken decades of research in the processes of particle synthesis and preparation [2], because, most often, they require to be further modified (or functionalized) to fit the desired application. This has been specially demonstrated in the case of the biomedical field [3]. Consider, for instance, the production of nanoparticle-based drug vehicles; these will find a series of obstacles in their way to the place of action [4], starting with the adsorption of plasma opsonins, which make the particles immediately recognized by the macrophages of the phagocyte mononuclear system [5], thus limiting their route along the blood stream. As is well known, one of the procedures to make them stealthy is coating them with a suitable polymer (poly(ethylenglycol) or PEG is the typical example [6], although other examples can be mentioned, such as dextran or chitosan [7,8]). In all cases, they offer a steric obstacle to opsonin adsorption. In the context of the present work, the charge of the coating layers can play a role in the opsonin–particle interaction, as well as in blood platelet aggregation and particle–cell interactions [9,10]. On top of this, the vehicle must be provided typically with additional functionalization to make it specific for the type of cancer cell. In summary, it is frequently necessary that the final colloid is coated with two or more layers, each with its particular role [11,12]. On a more physical basis, it is rather usual that coating with a single layer does not guarantee stability of the coating, as has been found in relation to the coating of magnetite particles [13]. Needless to say, there are many examples of biological entities where multilayers are present, such as bacteria, bacteriophages, anti-bacterial coatings, or even blood cells.

The topic of the paper is the consideration of the electrokinetic properties of this kind of nanostructure, with the aim of learning how their response to AC electric fields can be used to obtain information about the structure of the coated particles, regarding the charge, thickness, and stability of the layers. This has also been already considered by Duval et al. for both planar and spherical interfaces [14,15], and for either dc or AC fields, using a different approach.

**Figure 1:**
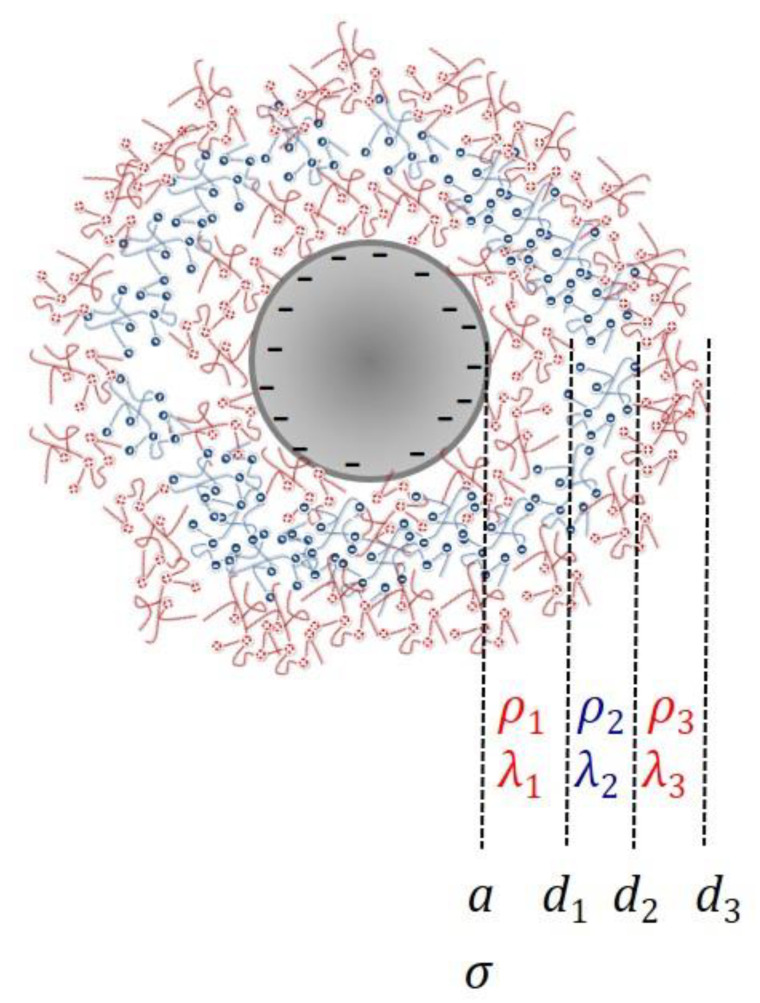
Schematic representation of a multi-layered soft particle (core radius: *a*; core surface charge density: σ; layer thickness: *d_i_*; layer volume charge density: ρ_i_; friction coefficient of the *i*-th layer: λ_i_).

For our purposes, the nanostructure is composed of a rigid particle (core) on which successive layers of charged (polyelectrolytes) or neutral polymers are deposited. Because the layers are typically both permeable to the solvent and the ions in solution, and deformable, the particles are often denominated “soft” [16]. The technique of deposition known as layer-by-layer [17,18] offers an excellent tool for producing such kinds of structures. Figure 1 is a representative scheme of the studied polymeric colloids.

In order to stress the role of the layering, the present study will be limited to the situation of so-called “salt-free” systems [19]. That is, ideally, our particles are coated by the polyelectrolyte and dispersed in water, without any additional salt; the only ions in solution (with the exception of H^+^, ${\mathrm{HCO}}_{3}^{-}$, and OH^−^ from water dissociation and atmospheric ${\mathrm{CO}}_{2}$. dissolution) are those released by the heterostructures upon charging. Experimentally, this situation will be approached using only water for the preparation of our suspensions. Interest in salt-free systems is also considerable; they can form long- or short-ranged ordered structures resembling crystalline solids, that is, colloidal crystals [20,21,22,23]. Their electrokinetic study was started and extended by Ohshima [24,25,26,27,28,29,30], although contributions by others must also be mentioned, particularly in the case of AC electric fields and concentrated suspensions [31,32,33,34,35].

Hence, the aims of this paper are as follows: (i) the elaboration of a model for the evaluation of the electrokinetics of multi-grafted polymer particles in the presence of alternating electric fields, in dispersion media where no salts are added; (ii) to carry out an experimental evaluation of the frequency dependence of the dynamic (or AC) electrophoretic mobility and the dielectric permittivity of suspensions of polystyrene latex spherical particles coated with successive layers of cationic, anionic, and neutral polymers; and (iii) finally, to perform a comparison between predictions and experimental results, so that it can be demonstrated that the electrokinetic analysis is a useful tool for the in situ characterization of multilayered particles.

## 2. Model

Let us consider a spherical particle of radius *a* as in Figure 1, immersed in a solution including the solvent (electric permittivity $\varepsilon_{s}\varepsilon_{0}$ and viscosity $\eta_{s}$) containing the counterions of the surface charge density σ of the particle and of the volume charge densities $\rho_{i}$ of the corresponding layers (the number of layers is $N_{l}$, $i=1,2,\ldots ,N_{l}$, three in the situation represented). The equilibrium electric potential $\Psi^{0}(r)$ at position **r** (the origin of the reference system is located in the particle centre) will be given by the Poisson–Boltzmann equation [36], with different charge densities, depending on the position (inside a polyelectrolyte layer or not). If $n_{j}^{0}(\infty )$ is the concentration (in number) of the *j*-th ionic species, and $z_{j}e$ is its charge, it can be written as follows:

(1) $$\begin{array}{c} \nabla^{2}\Psi^{0}(r)=0,\;r<a\\ \nabla^{2}\Psi^{0}(r)=-\frac{1}{\varepsilon_{s}\varepsilon_{0}}\sum_{j=1}^{N}ez_{j}n_{j}^{0}(\infty )\exp\left(-\frac{ez_{j}\Psi^{0}(r)}{k_{B}T}\right)-\frac{\rho_{1}}{\varepsilon_{s}\varepsilon_{0}},\;a<r<a+d_{1}\\ \nabla^{2}\Psi^{0}(r)=-\frac{1}{\varepsilon_{s}\varepsilon_{0}}\sum_{j=1}^{N}ez_{j}n_{j}^{0}(\infty )\exp\left(-\frac{ez_{j}\Psi^{0}(r)}{k_{B}T}\right)-\frac{\rho_{2}}{\varepsilon_{s}\varepsilon_{0}},\;a+d_{1}<r<a+d_{1}+d_{2}\\ \ldots \\ \nabla^{2}\Psi^{0}(r)=-\frac{1}{\varepsilon_{s}\varepsilon_{0}}\sum_{j=1}^{N}ez_{j}n_{j}^{0}(\infty )\exp\left(-\frac{ez_{j}\Psi^{0}(r)}{k_{B}T}\right),\;r>a+d_{1}+\ldots +d_{N_{l}} \end{array}$$

For the evaluation of the electrokinetics of these particles, it will be assumed that an alternating electric field with frequency ω is applied: $E=E_{0}\exp(-i\omega t)$. As a consequence, the equilibrium potential distribution will be modified, and the same will happen to all other quantities of interest. In particular, the fluid velocity **v** (relative to the particle, which moves with velocity **U** with respect to the laboratory frame), which is zero on average before the application of the field, will now obey the Navier–Stokes equations (incompressible fluid), in turn different for the different regions, as before:

(2) $$\begin{array}{c} \rho_{s}\frac{\partial \left(U+v\right)}{\partial t}=-\nabla p+\eta_{s}\nabla^{2}\left(U+v\right)-\sum_{j=1}^{N}ez_{j}n_{j}\nabla \Psi -\gamma_{1}v,\;a<r<a+d_{1}\\ \ldots \\ \rho_{s}\frac{\partial \left(U+v\right)}{\partial t}=-\nabla p+\eta_{s}\nabla^{2}\left(U+v\right)-\sum_{j=1}^{N}ez_{j}n_{j}\nabla \Psi ,\;r>a+d_{1}+\ldots +d_{N_{l}}\\ \nabla \cdot v=0 \end{array}$$

where the friction term $\gamma_{i}v$ is added inside the *i*-th layer. It is usually expressed relative to the fluid viscosity, yielding the quantity $\lambda_{i}{}^{2}=\gamma_{i}/\eta_{s}$, characteristic of the layer permeability. Note that this term comes from modelling the polymer layer as a set of spherical particles (the chains segments) uniformly distributed and producing Stokes friction. The conservation equation for ionic species will read as follows (Nernst–Planck equation):

(3) $$\begin{array}{l} \frac{\partial n_{j}}{\partial t}=-\nabla \cdot (n_{j}v_{j})\\ v_{j}=v-\frac{D_{j}}{k_{B}T}\nabla \mu_{j}\\ \mu_{j}=\mu_{j}^{\infty }+z_{j}e\Psi +k_{B}T\ln n_{j} \end{array}$$

where $\mu_{j}$ is the chemical potential ($\mu_{j}^{\infty }$ is its reference value) and $D_{j}$ is the diffusion coefficient of ions of type *j*. It is reasonable to assume that the applied field strength is low enough for the potential (and other quantities) to be perturbed by an amount linearly dependent on the field:

(4) $$\begin{array}{l} \Psi (r,t)=\Psi^{0}(r)+\delta \Psi (r,t)=\Psi^{0}(r)+\psi (r)E_{0}\cdot \hat{r}\exp(-i\omega t)\\ \mu_{j}(r,t)=\mu_{j}^{0}(r)+\delta \mu_{j}(r,t)=\mu_{j}^{0}(r)+\phi_{j}(r)E_{0}\cdot \hat{r}\exp(-i\omega t)\\ P(r,t)=P^{0}(r)+p(r)E_{0}\cdot \hat{r}\exp(-i\omega t) \end{array}$$

The final set of differential equations [37] can be obtained as follows:

(5) $$\begin{array}{c} L\left\{\psi (r)\right\}=\frac{e^{2}}{\varepsilon_{s}\varepsilon_{0}k_{B}T}\sum_{j=1}^{N}z_{j}^{2}n_{j}^{0}(r)\left[\phi_{j}(r)+\psi (r)\right]\\ L\left\{\phi_{j}(r)\right\}+\xi_{j}^{2}\left[\phi_{j}(r)+\psi (r)\right]=\frac{d\Psi^{0}(r)}{dr}\left[\frac{ez_{j}}{k_{B}T}\frac{d\phi_{j}}{dr}-\frac{2}{D_{j}}\frac{h(r)}{r}\right]\\ L\left\{L+\chi^{2}\right\}\left\{h(r)\right\}=\left\{ \begin{array}{ll} -\frac{e^{2}}{\eta k_{B}T}\frac{1}{r}\frac{d\Psi^{0}(r)}{dr}\sum_{j=1}^{N}z_{j}^{2}n_{j}^{0}(r)\phi_{j}(r)+\lambda_{i}Lh(r) & a+d_{1}+\ldots +d_{i-1}<r<a+d_{1}+\ldots +d_{i}\\ -\frac{e^{2}}{\eta k_{B}T}\frac{1}{r}\frac{d\Psi^{0}(r)}{dr}\sum_{j=1}^{N}z_{j}^{2}n_{j}^{0}(r)\phi_{j}(r) & r>a+d_{1}+\ldots +d_{N_{l}} \end{array}\right. \end{array}$$

In these equations, *L* is a differential operator, defined as follows:

(6) $$L\left\{\cdot \right\}=\frac{d^{2}\left\{\cdot \right\}}{dr^{2}}+\frac{2}{r}\frac{d\left\{\cdot \right\}}{dr}-\frac{2\left\{\cdot \right\}}{r^{2}}$$

and

(7) $$\begin{array}{l} \chi^{2}=\frac{i\omega \rho_{s}}{\kappa^{2}\eta_{s}}\\ \xi_{j}^{2}=\frac{i\omega }{D_{j}\kappa^{2}} \end{array}$$

where κ is the well-known Debye–Hückel parameter (reciprocal Debye length). The auxiliary function h(r) is the result of assuming spherical symmetry for the fluid velocity. Using spherical coordinates with origin in the particle centre, and the θ coordinate indicating the angle between the electric field and the position vector,

(8) $$v=\left[-2\frac{h(r)}{r}E\cos\theta ,\frac{1}{r}\frac{d}{dr}\left(rh(r)\right)E\sin\theta ,0\right]$$

The solids concentration by volume is ϕ and, if this is sufficiently high, hydrodynamic and electrical interactions between the particles must be taken into account. With that aim, the so-called cell model will be used. This was first introduced by Kuwabara [38], and further elaborated by different authors [24,35,39,40,41]. It is based on the assumption that the cited interactions can be represented by assuming that a single particle is in a concentric sphere of solution, such that its radius *b* guarantees that the volume fraction of solids in the sphere (the cell) is the same as in the whole dispersion:

(9) $$\phi ={\left(\frac{a}{b}\right)}^{3}$$

and the boundary conditions for the quantities of interest at r=b will determine the interactions.

The necessary boundary conditions come from the following:

(a) On the core particle surface:

- The equilibrium electric field on the surface must satisfy the Gauss law:
  (10) $$\sigma =-\varepsilon_{s}\varepsilon_{0}{\left(\frac{d\Psi^{0}}{dr}\right)}_{r=a}$$
- Impermeability to ions:
  (11) $${\left(\frac{d\phi_{j}}{dr}\right)}_{r=a}=0$$
- Stagnancy of the liquid:
  (12) $$h(a)=0,\;{\left(\frac{dh}{dr}\right)}_{r=a}=0$$
- Discontinuity of the normal component of the displacement vector ($\varepsilon_{p}$ is the relative permittivity of the particle). Using Equations (4) and (10), and the continuity of the potential,
  (13) $${\left(\frac{d\psi }{dr}\right)}_{r=a}-\frac{\varepsilon_{p}}{\varepsilon_{m}a}\psi (a)=0$$

(b) Between successive polyelectrolyte layers:

- Continuity of the potential and of the normal displacement vector:
  (14) $$\begin{array}{l} \psi {\left(a+\ldots +d_{i}\right)}^{-}=\psi {\left(a+\ldots +d_{i}\right)}^{+}\\ {\left(\frac{d\psi }{dr}\right)}_{{\left(a+\ldots +d_{i}\right)}^{-}}={\left(\frac{d\psi }{dr}\right)}_{{\left(a+\ldots +d_{i}\right)}^{+}} \end{array}$$
- Continuity of the normal and tangential components of the fluid velocity:
  (15) $$\begin{array}{l} h{\left(a+\ldots +d_{i}\right)}^{-}=h{\left(a+\ldots +d_{i}\right)}^{+}\\ {\left(\frac{dh}{dr}\right)}_{{\left(a+\ldots +d_{i}\right)}^{-}}={\left(\frac{dh}{dr}\right)}_{{\left(a+\ldots +d_{i}\right)}^{+}} \end{array}$$
- Continuity of the vorticity and the pressure ($\rho_{s}$ is the density of the solution):
  (16) $$\begin{array}{c} {\left[Lh\right]}_{{\left(a+\ldots +d_{i}\right)}^{-}}={\left[Lh\right]}_{{\left(a+\ldots +d_{i}\right)}^{+}}\\ {\left[\frac{d}{dr}\left(Lh+\frac{i\omega \rho_{s}}{\eta_{s}}h\right)-\lambda_{i}{}^{2}\left(\frac{dh}{dr}+\frac{h}{r}\right)\right]}_{{\left(a+\ldots +d_{i}\right)}^{-}}={\left[\frac{d}{dr}\left(Lh+\frac{i\omega \rho_{s}}{\eta_{s}}h\right)-\lambda_{i}{}^{2}\left(\frac{dh}{dr}+\frac{h}{r}\right)\right]}_{{\left(a+\ldots +d_{i}\right)}^{+}}\\ {\left[\frac{d}{dr}\left(Lh+\frac{i\omega \rho_{s}}{\eta_{s}}h\right)-\lambda_{N_{l}}{}^{2}\left(\frac{dh}{dr}+\frac{h}{r}\right)\right]}_{{\left(a+\ldots +d_{N_{l}}\right)}^{-}}={\left[\frac{d}{dr}\left(Lh+\frac{i\omega \rho_{s}}{\eta_{s}}h\right)\right]}_{{\left(a+\ldots +d_{N_{l}}\right)}^{+}}\;(\mathrm{outermost}\;\mathrm{layer}) \end{array}$$
- Continuity of ionic concentrations and velocities:
  (17) $$\begin{array}{l} \phi_{j}{\left(a+\ldots +d_{i}\right)}^{-}=\phi_{j}{\left(a+\ldots +d_{i}\right)}^{+}\\ {\left(\frac{d\phi_{j}}{dr}\right)}_{{\left(a+\ldots +d_{i}\right)}^{-}}={\left(\frac{d\phi_{j}}{dr}\right)}_{{\left(a+\ldots +d_{i}\right)}^{+}} \end{array}$$

(c) On the cell surface:

- Electroneutrality of the cell for the equilibrium:
  (18) $${\left(\frac{d\Psi^{0}}{dr}\right)}_{r=b}=0$$
- The macroscopic quantities are the result of averages of local quantities in the cell volume [42]:
  (19) $$\begin{array}{c} \psi (b)=-b\\ \phi_{j}(b)=b\\ p(b)=0 \end{array}$$

The problem can finally be solved by considering the equation of motion of the whole cell. Upon solving the differential equations, the quantities of interest can be calculated by evaluating the functions on the cell surface:

Electrophoretic mobility:

(20) $$u_{e}=\frac{2h(b)}{b}$$

Complex conductivity:

(21) $$K^{*}(\omega )={\left[\sum_{j=1}^{N}\frac{z_{j}e^{2}D_{j}}{k_{B}T}n_{j}^{0}\left(b\right)\frac{d\phi_{j}}{dr}\right]}_{b}-\sum_{j=1}^{N}z_{j}en_{j}^{0}(b)\frac{2h(b)}{b}+i\omega \varepsilon_{s}\varepsilon_{0}{\left(\frac{d\psi }{dr}\right)}_{b}$$

Relative permittivity ($\varepsilon^{*}=\varepsilon^{\prime }+i\varepsilon^{\prime \prime }$):

(22) ε′(ω)=−Im[K*(ω)]ωε0ε″(ω)=Re[K*(ω)]−Re[K*(ω→0)]ωε0

## 3. Materials and Methods

### 3.1. Materials

The polysterene latex spheres (1000 nm nominal diameter) were purchased from Ikerlat Polymers (Lasarte-Oria, Spain). According to the manufacturer, these particles have been extensively dialysed in water, and they have negative sulfate groups on their surface, yielding a surface charge density of −32 μC/cm^2^. All chemicals were from Sigma Aldrich (Darmstadt, Germany), and the water used in the preparation of the suspensions was deionized and filtered in a Milli-Q Academic (Millipore, Darmstadt, Germany) device.

### 3.2. Methods

The coating of the particles with the desired layer of the anionic polyelectrolyte poly(sodium 4-styrenesulfonate) (PSS, MW 70,000 g/mol), the cationic one Poly-(diallyldimethylammonium chloride) (PDADMAC, MW 200,000 g/mol), or the neutral polymer poly(ethylene oxide) (PEO, MW 900,000 g/mol) was carried out always using the same procedure: a suspension of spheres containing 2% (*w/v*) particles in a 100 mM solution of the polymer (calculated on a monomer basis) was kept under magnetic stirring for 24 h. Afterwards, the suspension was centrifuged (14,000 rpm, 10 min) and redispersed in water. This procedure was repeated three times to get rid of unadsorbed polymer. The final volume fractions of solids, ϕ, were as follows: 0.1 (bare polystyrene, PS); 0.036 (PS/PDADMAC); 0.054 (PS/PDADMAC/PSS); 0.033 (PS/PDADMAC/PSS/PDADMAC); 0.02 (PS/PDADMAC/PEO/PDADMAC).

The geometrical characteristics of the particles were determined by high-resolution transmission electron microscopy (HR-TEM, Auriga FIB-FESEM from Carl-Zeiss, Oberkochen, Germany). Electrophoretic mobilities were determined in a Zetasizer Nano-ZS (Malvern Instruments, Malvern, UK). 

**Figure 2:**
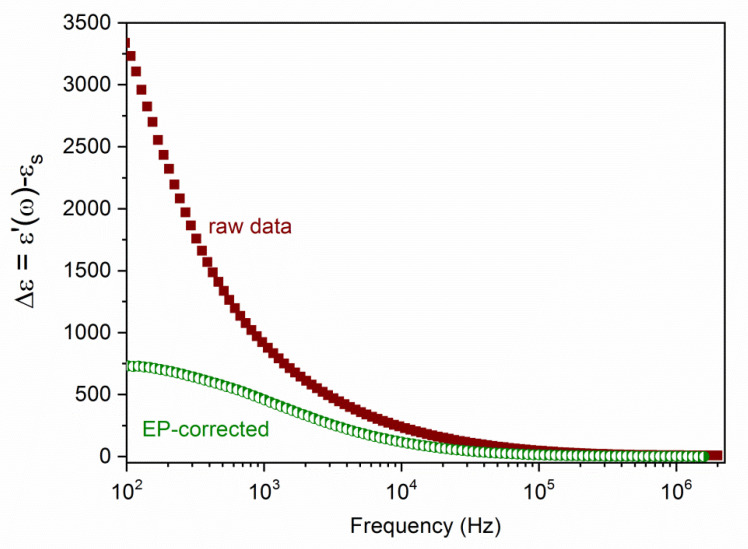
Dielectric increment (real part of the relative permittivity minus the solvent dielectric constant) as a function of frequency for untreated data (“raw”) and data corrected for electrode polarization (EP-corrected) by the logarithmic derivative method. System: 3.3% PS/PDADMAC/PSS/PDADMAC in water.

Dynamic (AC) electrophoretic mobilities were evaluated in the 2–18 MHz frequency range using a Colloidal Dynamics (USA) Acoustosizer IIc. For the complex conductivity or dielectric permittivity determinations, a lab-made cell (details in [43]) was used consisting of a thermostatted glass cylinder with parallel platinum electrodes (1 cm radius, 1 mm separation) connected to an Agilent E4980A (Santa Clara, CA, USA) LCZ meter, in the 1 kHz–2 MHz frequency interval. The cell was calibrated against KCl conductivity standards, for calculating the complex conductivity from the directly measured complex impedance $Z^{*}(\omega )$:

(23) $$K^{*}(\omega )=\frac{\lambda }{Z^{*}(\omega )}$$

where λ is the calibration cell constant. From this, the permittivity was determined, but an important step must be taken in order to eliminate (or, at least, minimize) the effect of electrode polarization, which can be a very important disturbing effect. Although different methods have been envisaged with this purpose, it was found that the most suitable one is based on calculating the logarithmic derivative of the raw $\varepsilon^{\prime }(\omega )$ data [43,44,45]. Figure 2 illustrates the efficiency of the method; the apparent high values of the dielectric constant at low frequencies are drastically reduced towards much more reasonable values after the treatment. This procedure was used for all the systems investigated.

## 4. Results and Discussion

### 4.1. Model Predictions

**Figure 3:**
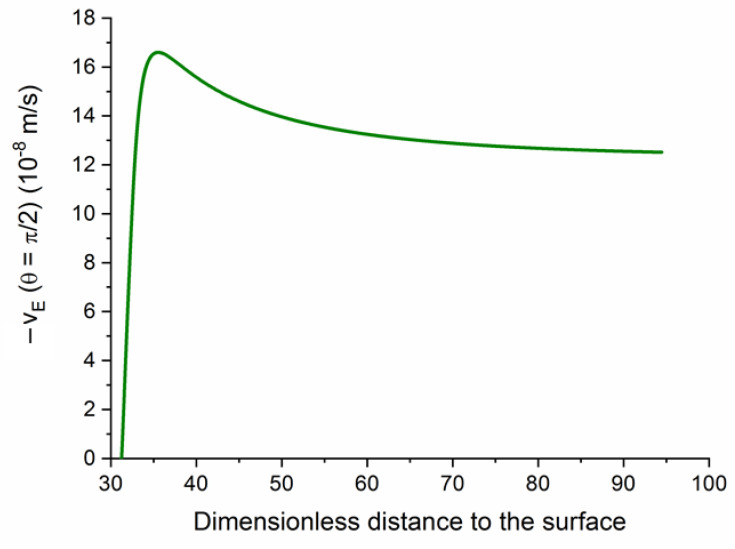
Profile of the component of the fluid velocity, relative to the particle, parallel to the external field, $v_{E}$ (for θ=π/2 ) for latex particles (1000 nm diameter and $-10{\;\mu C/\mathrm{cm}}^{2}$ surface charge density), coated by a single polyelectrolyte layer 40 nm thick, and charged with $+7\times 10^{5}{C/m}^{3}$. The distance is measured relative to the electrical double layer thickness of the core particle.

We will briefly show some predictions of the model above described. By way of example, Figure 3 shows the tangential (meaning parallel to the field, evaluated at θ=π/2) velocity profile as a function of the distance to the (negative) core surface for a single polyelectrolyte (cationic) layer. It is important to note that the fluid velocity grows steeply inside the layer, and the velocity on the layer limit determines the far-field value, and hence the electrophoretic velocity (equal and opposite to the latter; this is the practical reason for plotting minus the true velocity) of the particle.

**Figure 4:**
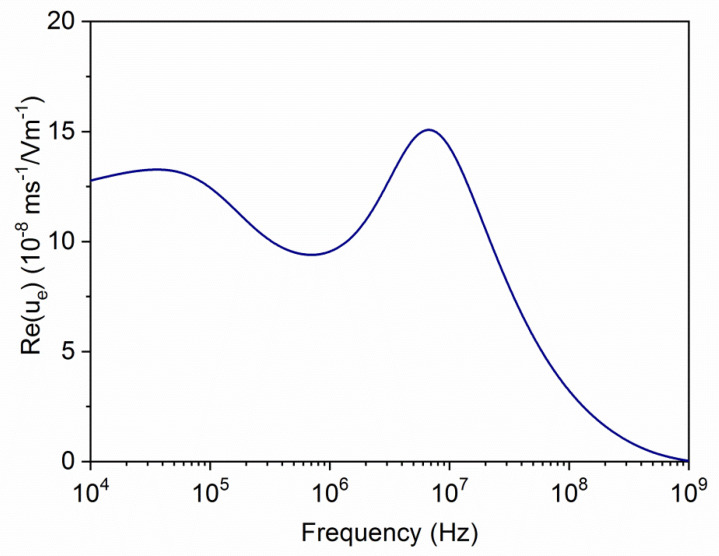
Real part of the dynamic mobility as a function of the field frequency for the same particle considered in Figure 3.

In Figure 4, the frequency dependence of the electrophoretic (dynamic) mobility for the same kind of particles is shown. Here, the main features of the colloid polarization are appreciated. Recall that the application of the alternating field (from left to right, say) produces different contributions to the electric polarization of the particle and its ionic atmosphere. Below the MHz range, the so-called Maxwell–Wagner–O’Konski (MWO) [46] polarization takes place; this is owing to the rearrangement of ions inside the layer: cations accumulate in the right side of the particle and anions in the opposite side, leading to a dipole parallel to the field. The characteristic dimension of the concentration perturbation is of the order of the layer thickness. If the frequency is raised above some characteristic value (MWO relaxation frequency), the ionic redistribution cannot occur, and the dipole moment is reduced. This gives rise to an increase in the mobility, as demonstrated in [46]. As frequency decreases, another mechanism is activated at the so-called alpha-relaxation frequency. This is related to the formation of a gradient of neutral electrolyte concentration at both sides of the particle owing to the different transport numbers of cations and anions inside the charged layer (the so-called concentration polarization [46,47]). In this mechanism, the characteristic dimension is comparable to the particle radius. The existence of concentration polarization has two counteracting effects: on one hand, the associated diffusive fluxes of ions reduce the strength of the MWO dipole, thus increasing the mobility; on the other hand, these diffusive fluxes convectively drag fluid in the charged region with them (this is the phenomenon of capillary osmosis), in the opposite direction of the electrophoretic mobility, which is thus reduced. Depending on the balance of the two mechanisms, the mobility will decrease (if the dipole moment mechanism is dominating) or increase (if capillary osmosis dominates) with frequency.

All these effects are observed in the predictions of Figure 4. In this case, the alpha-relaxation firstly produces an increase of *u_e_*, which can be related to the capillary osmosis inside the highly charged polyelectrolyte layer. When the frequency raises above 40 kHz, a mobility decline is predicted, which can be related to the concentration polarization of the oppositely charged core particle, which is acting on the induced dipole moment of the composite. At larger frequencies, the MWO rise is clearly observed and, finally, the subsequent decrease is a manifestation of the inertia of the fluid and the particle, leading to a tendency of the mobility to zero for sufficiently high frequencies.

**Figure 5:**
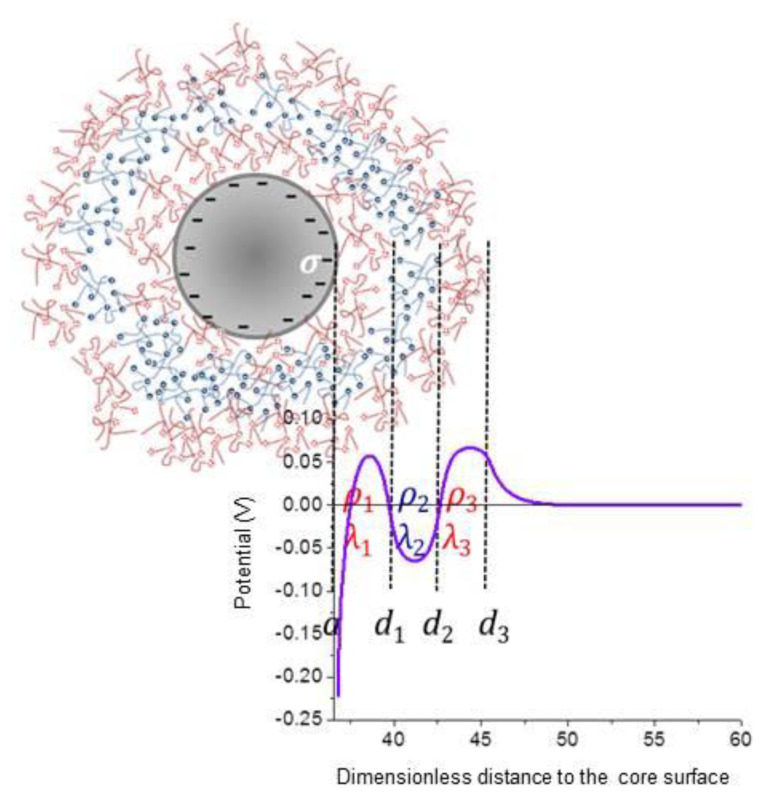
Electric potential vs. distance to the core surface for a triple polyelectrolyte layer. Calculations assuming that the core is a sphere 1000 nm in diameter and $-10{\;\mu C/\mathrm{cm}}^{2}$ surface charge, coated by successive layers of PDADMAC (40 nm $7\times 10^{5}{\;C/m}^{3}$ ), PSS (40 nm $-7\times 10^{5}{\;C/m}^{3}$ ), and PDADMAC (40 nm $7\times 10^{5}{\;C/m}^{3}$ ).

Let us now consider what can be expected when the particles are coated by a multilayer. Specifically, it will be assumed that the core (1000 nm in diameter) has a negative surface charge of $-10{\;\mu C/\mathrm{cm}}^{2}$, and that the successive polymer layers are alternatively positive ($7\times 10^{5}{\;C/m}^{3}$), negative ($-7\times 10^{5}{\;C/m}^{3}$), and positive ($7\times 10^{5}{\;C/m}^{3}$), each 40 nm thick. Figure 5 illustrates the potential versus distance relationship for this case. The gradual variation of the potential, the changing sign according to the charge of the layer, as well as the existence of a region of approximately constant potential inside each of the polymer regions can be observed, corresponding to their respective Donnan potentials [48,49]. Finally, the decay to zero after the outermost layer is a manifestation of the existence of the electrical double layer between the polyelectrolyte and the medium.

**Figure 6:**
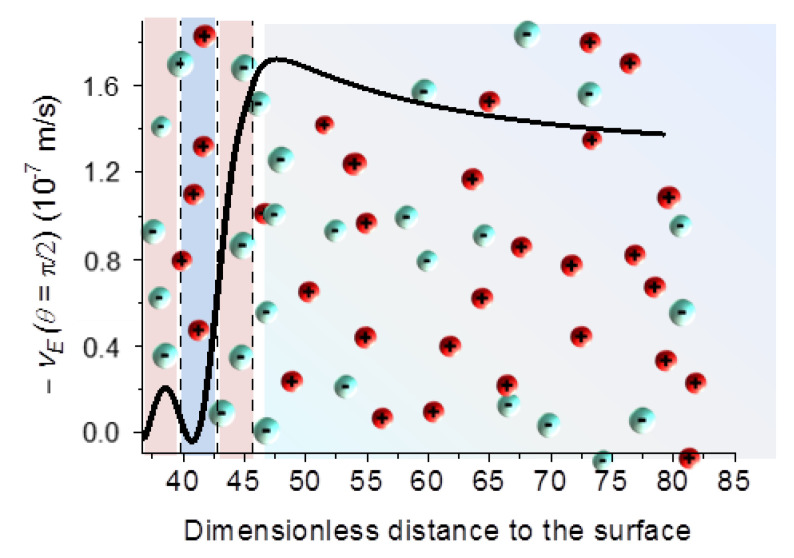
Tangential velocity profile (parallel to the external field, θ=0) for the case shown in Figure 5.

The tangential fluid velocity profile displayed in Figure 6 is also a good indication of the physics of the electrokinetics in these systems. For the same particles considered in Figure 5 and Figure 6 shows how the fluid velocity changes through the triple layer, yielding a final (far field as before) velocity determined in this case by the outer, positive layer. This is a significant warning regarding how far the measurement of a single DC electrophoretic mobility is from providing information about the nanostructure of the core/shell particle. In contrast, as will be shown below, AC electrokinetics data provided with a suitable theoretical model can give us very useful details.

**Figure 7:**
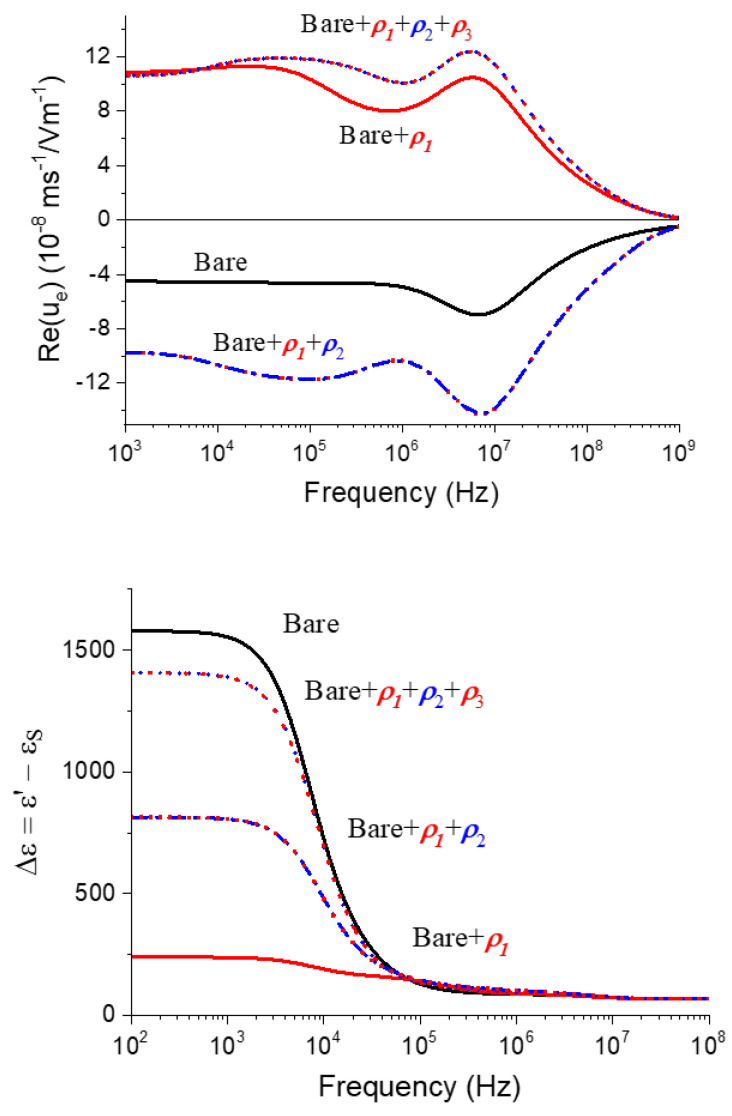
Theoretical predictions of the real part of the dynamic mobility (top) and dielectric dispersion (bottom) for 2% suspensions of latex particles with the coatings indicated. The core particle is a 1000 nm diameter polystyrene particle with $-10\;{\mu C/\mathrm{cm}}^{2}$ surface charge. All coatings are 40 nm thick, and their volume charges are as follows (in C/m^3^): $\rho_{1}=+7\times 10^{5},\;\rho_{2}=-7\times 10^{5},\;\rho_{3}=+7\times 10^{5}$.

Examples are provided in Figure 7, showing the frequency dependence of the mobility (real part) and the dielectric constant. Concerning the former, it is clear that the mobility spectrum changes (and not only in sign, as in principle expected) when the bare particles are coated with successive layers. The presence of the first positive coating (“bare + $\rho_{1}$”) corresponds to the situation described in detail in Figure 4. Of course, the method allows distinction between the positive coating owing to just one layer and by three of them (+/−/+: $\rho_{1}+\rho_{2}+\rho_{3}$). Thus, in the latter case, the mobility reduction associated with the alpha-relaxation of the core is less significant because it is hidden by a thicker polymer layer than in the case of “bare + $\rho_{1}$”. In contrast, the MWO elevation is practically unaffected, as the outer layer is the same in both cases. Regarding the frequency dependence of the dielectric constant of the latex suspensions, the predictions in Figure 7 demonstrate that this electrokinetic method sweeps a frequency range different (complementary, one could say) than dynamic electrophoresis. An intense relaxation is observed at frequencies around 10 kHz, which is related to the effect of concentration polarization on the induced dipole moment (the dielectric response is most sensitive to changes in the induced dipole moment, and it is much less affected by either capillary osmosis or MWO processes). Note, however, that in the case of “bare + $\rho_{1}$” particles, a hump in the dielectric dispersion is barely appreciated at the same frequency as the mobility reduction in this system. This hump is not visible for the other systems, in agreement with its less important role already observed in the mobility.

### 4.2. Particle Characterization

**Figure 8:**
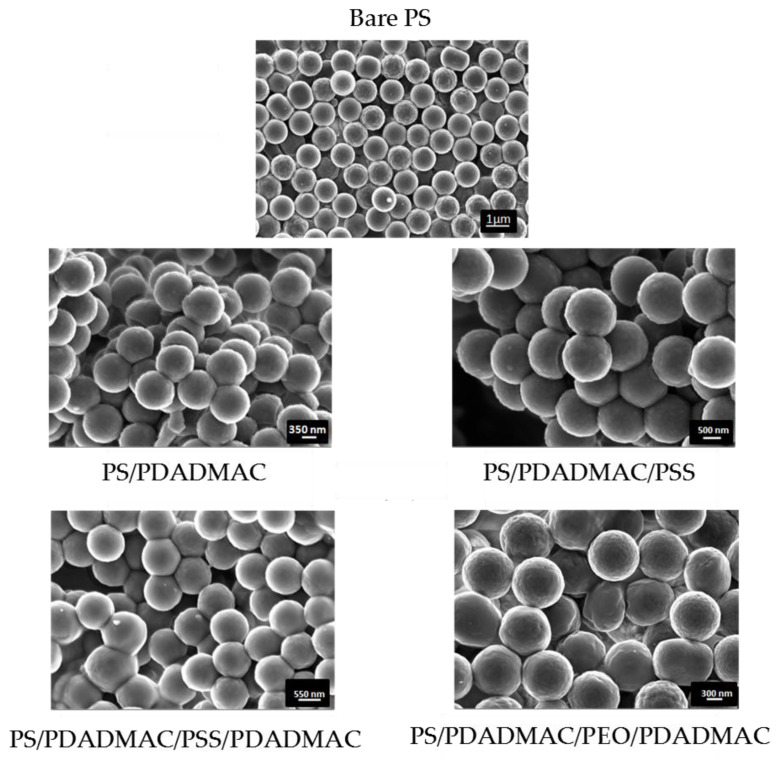
High-resolution transmission electron microscopy (HR-TEM) pictures of the bare and coated latex particles.

The particles were observed in all cases by HR-TEM, as described. Pictures of the latex spheres, both bare and coated with different layers, are shown in Figure 8. Note the sphericity and monodispersity of all the systems. In some of the pictures, the presence of incompletely coated particles can even help us in distinguishing the presence of the shell and its dimension. Table 1 includes the average and standard deviation of the radii of all the systems (measured on about 100 particles).

**Figure 9:**
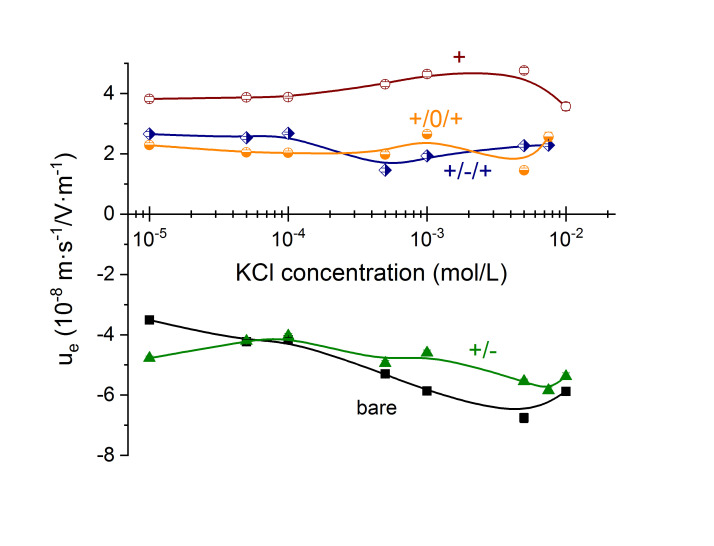
Electrophoretic mobility (DC field) as a function of KCl concentration for the particle systems studied. Points: experimental data. Lines a guide to the eye.

A preliminary electrical characterization was also carried out by determining the standard dc electrophoretic mobility of the particles as a function of KCl concentration. The results are presented in Figure 9. As expected, the mobility shows mainly the changes in the sign of the external layer of the particles as successive coatings are applied, but not much more information can be obtained. It is perhaps worthwhile to mention that the mobility of the bare particles increases (in absolute value) when the electrolyte concentration is increased up to 5 mM. This is contrary to the typical result of zeta potential (and mobility) reduction by electric double layer compression [50], but it has been repeatedly ascribed to the presence of a highly conductive Stern layer, typical of highly charged particles as the present latex [36,50]. The effect is hidden by the presence of the soft layers; in fact, when three highly charged layers are applied to the particles, the mobility is essentially independent of ionic strength, a fact discussed theoretically by Ohshima [24]. It is also noticeable that the results obtained with combinations +/−/+ and +/0/+ are practically indistinguishable, so that the presence of the uncharged intermediate layer does not affect the outer positive coating.

### 4.3. Dynamic Mobility and Permittivity of the Suspensions. Predictions and Experiments

**Figure 10:**
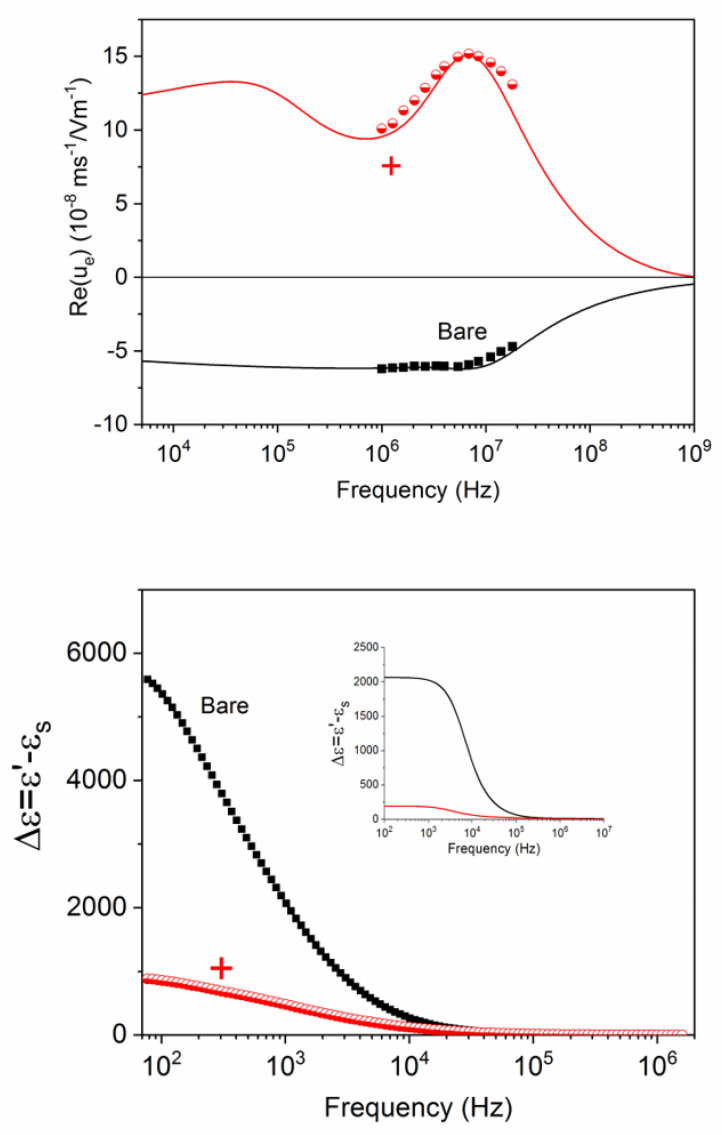
Top: real part of the dynamic mobility of bare and PDADMAC-coated (“+” in the plot) latex particles as a function of the field frequency; bottom: dielectric increment (dielectric constant relative to that of the solvent) for the same systems; inset: theoretical predictions using the parameters in Table 2. The symbols are experimental data, and the lines are fittings obtained with the model described.

Figure 10 (top) shows the effect of the addition of the first PDADMAC layer on the dynamic mobility of latex particles (the bare ones are included for comparison). For the calculations, a base KCl concentration was used to mimic the ionic strength of the particles counterions. The best results were obtained if the concentration (in millimole/L) was 10 ϕ. Table 2 shows the best-fit parameters used for obtaining the theoretical lines in these and the other cases. Note that the mobility is controlled by the outermost layer, as predicted by the model, and that the latter describes in great detail the behaviour of the mobility in the experimentally accessible frequency range. The MWO mobility elevation, more significant in the presence of the highly charged layer, is clearly observable and also well represented by our predictions.

With the fitting parameters, the dielectric dispersion of the suspensions can be predicted, and its comparison with the experimental results is plotted in Figure 10 (bottom). It is important to point out that the dielectric constant, in contrast with the mobility, is mostly sensitive to the alpha relaxation, which is well observed in both the bare and coated particles. The amplitude of the relaxation is, however, smaller in the latter case, indicating that such amplitude is roughly given by the overall charge of the particle, whether on its surface or in its polymer layer. Equally important, the theoretical predictions, although qualitatively very similar to the measured dielectric constant, are typically below these—a result very often reported, and probably a consequence of the existence of a conductive Stern layer, referred to above [51,52].

**Figure 11:**
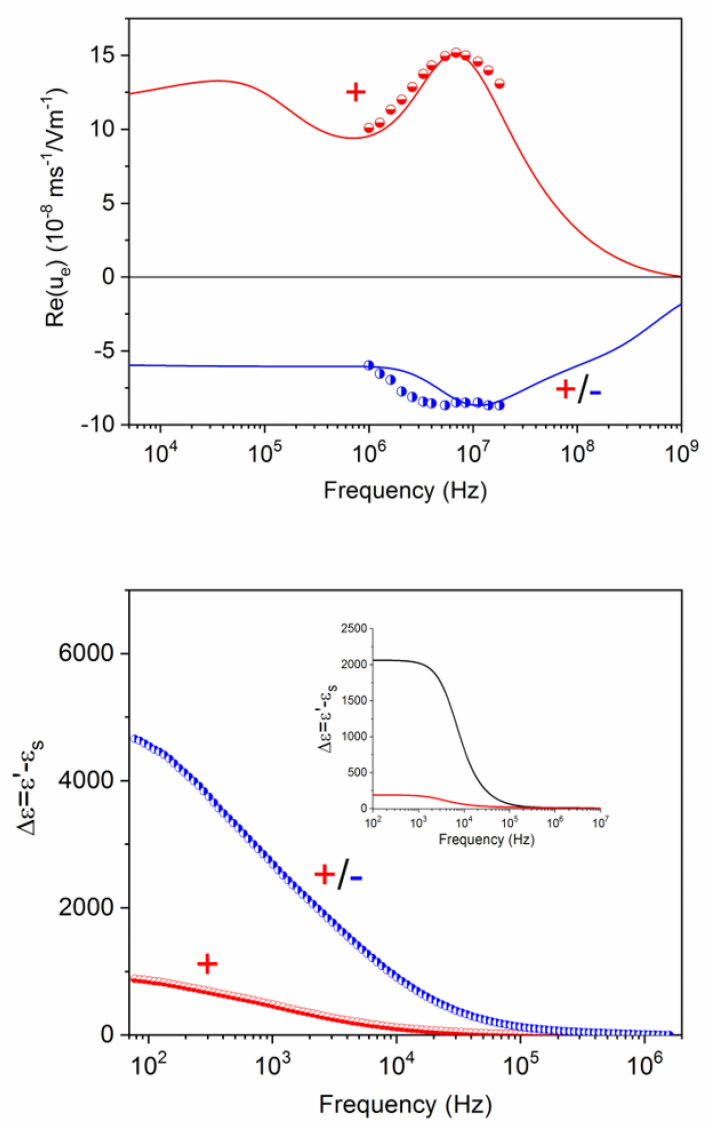
Same as Figure 10, but for latex particles coated with PDADMAC (+) and PDADMAC/PSS (+/−).

If a second layer (negative, constituted by PSS) is added to the system in Figure 10, the results obtained are illustrated in Figure 11. The negative sign of the dynamic mobility is again a proof of the determinant role of the external coating on the particle velocity. The agreement between theory and experiment is also clear, if the parameters in Table 2 are used for the predictions. There is, however, a certain disagreement regarding the position in frequency of the MWO maximum, lower in the case of the experiment (Figure 11 top). This gives us new information on the nanostructure of the layers; in fact, it could be explained by assuming a partial overlap of the + and − layers; the corresponding MWO elevation will be shifted to lower frequencies, as a consequence of the partial neutralization of the charges of the two layers. In favour of these arguments is the finding that the best-fit value of the charge of the PDADMAC layer is lower in the +/− than in the purely positive coating. Interestingly, this phenomenon is as well suggested by the permittivity data in Figure 11, bottom. The dielectric relaxation of the +/− particles extends over a wider frequency range than that of the positively coated particles. This indicates a merging of the alpha and MWO relaxations. A coherent picture is also provided by the fact that the dielectric amplitude grows again when the negative layer is added; the total charge (including that the core particle) increases again, no matter with which sign.

**Figure 12:**
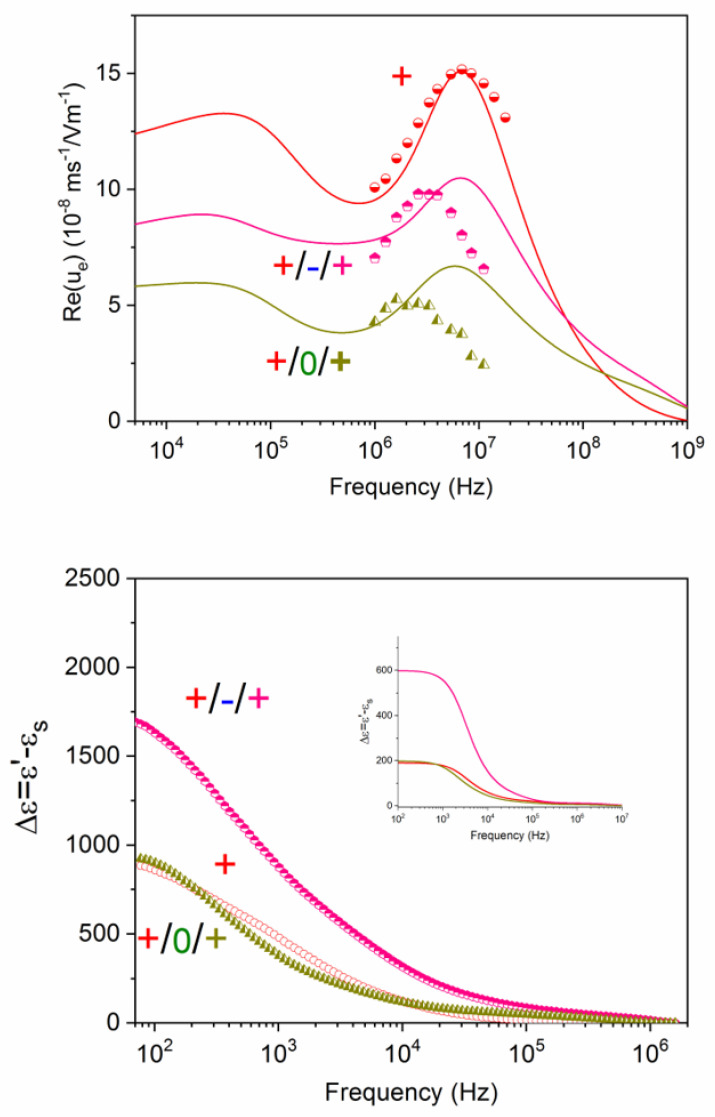
Real part of the dynamic mobility (top) and dielectric increment (bottom) of suspensions of polystyrene particles coated by PDADMAC (+), PDADMAC/PSS/PDADMAC (+/−/+), and PDADMAC/PEO/PDADMAC (+/0/+).

Finally, let us consider the more complex situation of triple layers, either +/−/+ or +/0/+, as shown in Figure 12, where the positive single layer (+) is included as reference. The whole set of data gives us important clues as to the structure of the layers, although also in this case a single mobility or dielectric spectrum would be insufficient for providing such information. The help of the model predictions and of the information obtained from the simpler structures above described can help us in reaching a coherent description. Note first of all that the frequency dependencies of the mobility of the three kinds of positive particles are clearly different, beyond the expected fact that the mobilities are positive for the whole frequency range. The mobility of the particles coated with just one positive layer is the highest of the three systems: the lowest value is, in contrast, associated to the neutral intermediate layer. It is suggested that the positive coating is more efficient if the substrate is the (highly negatively charged) bare particle or the negative PSS shell. As a consequence, when the intermediate polymer is PEO, the MWO mobility elevation is less significant, suggesting inhomogeneous or weaker attachment of the outermost positive layer. The permittivity data (Figure 12 bottom), leaving aside the quantitative differences with the predictions, are compatible with a better positive coating in the +/−/+ case: thus, the dielectric amplitude is larger for +/−/+ than for +/0/+ systems. Note, additionally, that there is almost no difference between purely positive and +/0/+. However, the decay is somewhat faster in the latter case, suggesting a more compact layer, consequence of the mentioned layer superposition.

## 5. Limitations and Future Scope of the Model

The model intends to be quite general, and applicable to arbitrary values of particle size, charge and concentration, and number and charge of layers. Improvements can be expected regarding the possibility that the layers can be interpenetrated, so that neighbour layers diffuse into each other. In addition, it should be considered that the layers can be only partially ionized, and a fraction of their counterions may remain immobilized close to their sites, forming a counterion condensate [53]. Knowledge of these facts might avoid that the charge of the layers and the counterion concentrations are fitting parameters, as in the present approach. As a result of these improvements, it should be possible to obtain a great deal of information on the polyelectrolyte coating structure with just dynamic mobility data, which are quite easily accessible.

## 6. Conclusions

In this work, it is shown that it is possible to modify the nanostructure of the polymer solution interface in the case of polystyrene particles, by coating them with different combinations of positive (PDADMAC), negative (PSS), or neutral (PEO) layers, using the layer-by-layer technique. In contrast with traditional methods such as dc electrophoresis, it is proposed that an electrokinetic study using AC techniques, namely dynamic (ac) electrophoresis and dielectric dispersion of the suspensions, can be a really informative tool about the multilayer structure. With the purpose of obtaining information about the latter (charge, thickness, and friction coefficient of each layer), a general model was elaborated for the dynamic mobility and dielectric constant of suspensions of spheres with arbitrary soft coatings and concentrations of solids. The model is capable of predicting the AC electrokinetics of the systems, and demonstrates to be very sensitive to the layer configuration. It is used for interpreting the experimental results obtained with latex particles coated with PDADMAC (+), PDADMAC/PSS (+/−), PDADMAC/PSS/PDADMAC (+/−/+), and PDADMAC/PEO/PDADMAC (+/0/+). The dielectric constant (1 kHz to 2 MHz) and dynamic electrophoretic mobility (2 to 18 MHz) of suspensions of 1000 nm polystyrene spheres with such coatings were determined at 25 °C. It was found that the mobility is controlled by the outermost layer regarding the overall sign, although the amplitude and frequency extent of concentration polarization and Maxwell–Wagner–O’Konski relaxations differ for a single layer and multiple layers. The dielectric permittivity is found to undergo intense relaxations at a low (kHz) frequency (alpha process), associated with the concentration polarization phenomenon. This is mostly controlled by the total charge of the particle because, the larger the overall charge of the nanostructure, the higher the dielectric amplitude (the decay in the dielectric constant). There is, however, some effect on the overall decay rate, and in some cases, the presence of a low-frequency MWO is barely appreciated. The agreement with the model is mostly qualitative in the case of dielectric dispersion, and yields quantitatively valid predictions on dynamic mobility. Jointly considered, the data demonstrate the versatility of AC electrokinetics and the amount of information that can be obtained from it if a suitable model is available, such as the one elaborated in this work.

## Figures and Tables

**Table 1:** Average (±S.D.) particle diameters of the latex particles investigated.

Particle	Diameter (±S.D.) (nm)
Bare PS	1000 ± 60
PS/PDADMAC (+)	1060 ± 60
PS/PDADMAC/PSS (+/−)	1240 ± 60
PS/PDADMAC/PSS/PDADMAC (+/−/+)	1060 ± 100
PS/PDADMAC/PEO/PDADMAC (+/0/+)	1120 ± 100

**Table 2:** Best-fit parameters of the model, obtained from experimental mobility data.

Particle	Layer Thickness (nm)	Layer Charge (C/m^3^)	$\mathrm{Dimensionless}\;\mathrm{Friction}\;\mathrm{Parameter}\;\lambda_{i}a$
PS/PDADMAC (+)	40	7 × 10^5^	10
PS/PDADMAC/PSS (+/−)	50/50	1 × 10^5^/−7 × 10^5^	10/35
PS/PDADMAC/PSS/PDADMAC (+/−/+)	50/50/40	1 × 10^5^/−1 × 10^5^/7 × 10^5^	10/35/25
PS/PDADMAC/PEO/PDADMAC (+/0/+)	40/2/10	7 × 10^5^/0/7 × 10^5^	30/35/30

## References

[1] Stark W.J., Stoessel P.R., Wohlleben W., Hafner A. (2015). Industrial applications of nanoparticles. Chem. Soc. Rev..

[2] Kestell A.E., DeLorey G.T. (2009). Nanoparticles: Properties, Classification, Characterization.

[3] Delgado A.V., López-Viota J., Ramos-Tejada M.M., Arias J.L., Oshima H. (2014). Particle geometry, charge, and wettability: The fate of nanoparticle-based drug vehicles. Colloid and Interface Science in Pharmaceutical Research and Development.

[4] Petros R.A., DeSimone J.M. (2010). Strategies in the design of nanoparticles for therapeutic applications. Nat. Rev. Drug Discov..

[5] Hume D.A. (2006). The mononuclear phagocyte system. Curr. Opin. Immunol..

[6] Howard M.D., Jay M., Dziublal T.D., Lu X. (2008). PEGylation of nanocarrier drug delivery systems: State of the art. J. Biomed. Nanotechnol..

[7] Deng J., He J., Zheng J.-S., Terakawa S., Huang H., Fang L.-C., Li Y., Cheng P., Jiang L.-L. (2013). Preparation and Application of Amino- and Dextran-Modified Superparamagnetic Iron Oxide Nanoparticles. Part. Sci. Technol..

[8] Gulbake A., Jain S.K. (2012). Chitosan: A potential polymer for colon-specific drug delivery system. Expert Opin. Drug Deliv..

[9] Dobrovolskaia M.A., Aggarwal P., Hall J.B., McNeil S.E. (2008). Preclinical studies to understand nanoparticle interaction with the immune system and its potential effects on nanoparticle biodistribution. Mol. Pharm..

[10] Canton I., Battaglia G. (2012). Endocytosis at the nanoscale. Chem. Soc. Rev..

[11] Pietronave S., Iafisco M., Locarno D., Rimondini L., Prat M. (2009). Functionalized nanomaterials for diagnosis and therapy of cancer. J. Appl. Biomater. Biomech..

[12] Allen T.M. (2002). Ligand-targeted therapeutics in anticancer therapy. Nat. Rev. Cancer.

[13] Del Mar Ramos-Tejada M., Viota J.L., Rudzka K., Delgado A.V. (2015). Preparation of multi-functionalized Fe3O4/Au nanoparticles for medical purposes. Colloids Surf. B Biointerfaces.

[14] Merlin J., Duval J.F.L. (2014). Electrodynamics of soft multilayered particles dispersions: Dielectric permittivity and dynamic mobility. Phys. Chem. Chem. Phys..

[15] Duval J.F.L., Werner C., Zimmermann R. (2016). Electrokinetics of soft polymeric interphases with layered distribution of anionic and cationic charges. Curr. Opin. Colloid Interface Sci..

[16] Ohshima H. (2009). Theory of electrostatics and electrokinetics of soft particles. Sci. Technol. Adv. Mater..

[17] Decher G., Hong J.D., Schmitt J. (1992). Buildup of ultrathin multilayer films by a self-assembly process. 3. consecutively alternating adsorption of anionic and cationic polyelectrolytes on charged surfaces. Thin Solid Films.

[18] Decher G. (1997). Fuzzy nanoassemblies: Toward layered polymeric multicomposites. Science.

[19] Oosawa F. (1963). Thermodynamic properties of rodlike polyelectrolyte solutions in presence of salts. J. Polym. Sci. A.

[20] Medebach M., Palberg T. (2003). Phenomenology of colloidal crystal electrophoresis. J. Chem. Phys..

[21] Wette P., Schope H.J., Palberg T. (2003). Experimental determination of effective charges in aqueous suspensions of colloidal spheres. Colloids Surf. A Physicochem. Eng. Asp..

[22] Medebach M., Palberg T. (2004). Electrophoretic mobility of electrostatically interacting colloidal spheres. J. Phys. Condens. Matter.

[23] Palberg T., Medebach M., Garbow N., Evers M., Fontecha A.B., Reiber H., Bartsch E. (2004). Electrophoresis of model colloidal spheres in low salt aqueous suspension. J. Phys. Condens. Matter.

[24] Ohshima H. (1994). Electrophoretic mobility of soft particles. J. Colloid Interface Sci..

[25] Ohshima H. (2002). Electrophoretic mobility of a spherical colloidal particle in a salt-free medium. J. Colloid Interface Sci..

[26] Ohshima H. (2003). Dynamic electrophoretic mobility of spherical colloidal particles in a salt-free medium. J. Colloid Interface Sci..

[27] Ohshima H. (2003). Electrokinetic phenomena in a dilute suspension of spherical colloidal particles in a salt-free medium. Colloids Surf. A Physicochem. Eng. Asp..

[28] Dukhin S.S., Zimmermann R., Werner C. (2006). Electrokinetic fingerprinting of grafted polyelectrolyte layers—A theoretical approach. Adv. Colloid Interface Sci..

[29] Duval J.F.L., Ohshima H. (2006). Electrophoresis of diffuse soft particles. Langmuir.

[30] Ohshima H. (2007). Electrokinetics of soft particles. Colloid Polym. Sci..

[31] Arroyo F.J., Carrique F., Ruiz-Reina E., Delgado A.V. (2011). Double layer polarization in “realistic” aqueous salt-free suspensions. Colloids Surf. A Physicochem. Eng. Asp..

[32] Roa R., Carrique F., Ruiz-Reina E. (2011). Electric double layer for spherical particles in salt-free concentrated suspensions including ion size effects. Phys. Chem. Chem. Phys..

[33] Vissers T., Imhof A., Carrique F., Delgado A.V., van Blaaderen A. (2011). Electrophoresis of concentrated colloidal dispersions in low-polar solvents. J. Colloid Interface Sci..

[34] Roa R., Carrique F., Ruiz-Reina E. (2012). Ion size effects on the electrokinetics of salt-free concentrated suspensions in ac fields. J. Colloid Interface Sci..

[35] Delgado A.V., Carrique F., Roa R., Ruiz-Reina E. (2016). Recent developments in electrokinetics of salt-free concentrated suspensions. Curr. Opin. Colloid Interface Sci..

[36] Lyklema J. (1995). Fundamentals of Interface and Colloid Science.

[37] Ahualli S., Jimenez M.L., Carrique F., Delgado A.V. (2009). AC Electrokinetics of Concentrated Suspensions of Soft Particles. Langmuir.

[38] Kuwabara S. (1959). The forces experienced by randomly distributed parellel circular cylinders or spheres in a viscous flow at small reynolds numbers. J. Phys. Soc. Jpn..

[39] Ohshima H. (1997). Dynamic electrophoretic mobility of spherical colloidal particles in concentrated suspensions. J. Colloid Interface Sci..

[40] Carrique F., Ruiz-Reina E., Roa R., Arroyo F.J., Delgado A.V. (2015). General electrokinetic model for concentrated suspensions in aqueous electrolyte solutions: Electrophoretic mobility and electrical conductivity in static electric fields. J. Colloid Interface Sci..

[41] Ahualli S., Gonzalez M.A., Delgado A.V., Jimenez M.L. (2017). Dynamic electrophoretic mobility and electric permittivity of concentrated suspensions of plate-like gibbsite particles. J. Colloid Interface Sci..

[42] Ahualli S., Delgado A., Miklavcic S.J., White L.R. (2006). Dynamic electrophoretic mobility of concentrated dispersions of spherical colloidal particles. On the consistent use of the cell model. Langmuir.

[43] Tirado M.C., Arroyo F.J., Delgado A.V., Grosse C. (2000). Measurement of the low-frequency dielectric properties of colloidal suspensions: Comparison between different methods. J. Colloid Interface Sci..

[44] Jimenez M.L., Arroyo F.J., van Turnhout J., Delgado A.V. (2002). Analysis of the dielectric permittivity of suspensions by means of the logarithmic derivative of its real part. J. Colloid Interface Sci..

[45] Chassagne C., Dubois E., Jimenez M.L., van der Ploeg J.P.M., van Turnhout J. (2016). Compensating for Electrode Polarization in Dielectric Spectroscopy Stuides of Colloidal Suspensions: Theoretical Assessment of Existing Methods. Front. Chem..

[46] Shilov V.N., Delgado A.V., Gonzalez-Caballero E., Horno J., Lopez-Garcia J.J., Grosse C. (2000). Polarization of the electrical double layer. Time evolution after application of an electric field. J. Colloid Interface Sci..

[47] Dukhin S.S., Shilov V.N. (1974). Dielectric Phenomena and the Double Layer in Disperse Systems and Polyelectrolytes.

[48] Ohshima H. (2012). Electrical phenomena in a suspension of soft particles. Soft Matter.

[49] Ohshima H. (2013). Electrokinetic phenomena of soft particles. Curr. Opin. Colloid Interface Sci..

[50] Hunter R.J. (1987). Foundations of Colloid Science.

[51] Arroyo F.J., Carrique F., Bellini T., Delgado A.V. (1999). Dielectric dispersion of colloidal suspensions in the presence of stern layer conductance: Particle size effects. J. Colloid Interface Sci..

[52] Carrique F., Arroyo F.J., Delgado A.V. (2002). Electrokinetics of concentrated suspensions of spherical colloidal particles with surface conductance, arbitrary zeta potential, and double-layer thickness in static electric fields. J. Colloid Interface Sci..

[53] Jimenez M.L., Delgado A.V., Ahualli S., Hoffmann M., Wittemanb A., Ballauff M. (2011). Giant permittivity and dynamic mobility observed for spherical polyelectrolyte brushes. Soft Matter.

